# Supplementary material for: Role of Continuous Glucose Monitoring in Supporting Glycemic Control Among Adolescents with Type 1 Diabetes in Saudi Arabia: A Retrospective Study
Source: Healthcare (Basel). 2025 Feb 25;13(5):496. doi: 10.3390/healthcare13050496 (PMC11898648; doi:10.3390/healthcare13050496)
Supplement: Supplementary file 1 [file healthcare-13-00496-s001.zip › healthcare-3483555-supplementary.pdf]

STROBE Checklist for Observational Studies

This checklist is based on the Strengthening the Reporting of Observational Studies in Epidemiology (STROBE) guidelines. Please complete this checklist by indicating whether each item has been addressed in your manuscript.

Section	STROBE Item	Addressed in Manuscript (Yes/No/Page No.)
Title and Abstract	1. Indicate the study design with a commonly used term in the title or the abstract.	Yes/ Page No 1
Introduction	2. Background/rationale: Explain the scientific background and rationale for the investigation.	Yes/ Page No 2
Introduction	3. Objectives: State specific objectives, including prespecified hypotheses.	Yes/ Page No 2
Methods	4. Study design: Present key elements of study design early in the paper.	Yes/ Page No 3
Methods	5. Setting: Describe the setting, locations, and relevant dates, including periods of recruitment, exposure, follow-up, and data collection.	Yes/ Page No 3
Methods	6. Participants: Give the eligibility criteria, and the sources and methods of selection of participants.	Yes/ Page No 2
Methods	7. Variables: Clearly define all outcomes, exposures, predictors, potential confounders, and effect modifiers.	Yes/ Page No 3
Methods	8. Data sources/measurement: For each variable of interest, describe data sources and measurement methods.	Yes/ Page No 4
Methods	9. Bias: Describe any efforts to address potential sources of bias.	Yes/ Page No 2
Methods	10. Study size: Explain how the study size was determined.	Yes/ Page No 2

Methods	11. Quantitative variables: Explain how quantitative variables were handled in the analyses.	Yes/ Page No 4
Methods	12. Statistical methods: Describe all statistical methods, including those used to control for confounding.	Yes/ Page No 4
Results	13. Participants: Report the number of individuals at each stage of study.	Yes/ Page No 5
Results	14. Descriptive data: Give characteristics of study participants.	Yes/ Page No 5
Results	15. Outcome data: Report numbers of outcome events or summary measures.	Yes/ Page No 6
Results	16. Main results: Report the main study findings and effect estimates.	Yes/ Page No 6
Results	17. Other analyses: Report other analyses done, such as sensitivity analyses.	Yes/ Page No 8
Discussion	18. Key results: Summarize key results with reference to study objectives.	Yes/ Page No 9
Discussion	19. Limitations: Discuss limitations of the study, taking into account sources of bias and imprecision.	Yes/ Page No 10
Discussion	20. Interpretation: Give a cautious overall interpretation of results.	Yes/ Page No 10
Discussion	21. Generalizability: Discuss the generalizability (external validity) of the study findings.	Yes/ Page No 10
Other Information	22. Funding: Give the source of funding and role of the funders if applicable.	Yes/ Page No 11

Note: This checklist is provided to ensure transparency in observational research reporting. Refer to the STROBE guidelines (DOI: 10.1016/j.jclinepi.2007.11.008) for further details.
